# Evaluating the Knowledge Level, Practice, and Behavioral Change Potential of Care Managers in Pressure Injury Prevention Using a Mobile App Prototyping Model in the Home-Care Setting: Single-Arm, Pre-Post Pilot Study

**DOI:** 10.2196/57768

**Published:** 2025-02-07

**Authors:** Masushi Kohta, Mayumi Takahashi, Hiroe Koyanagi, Junko Sugama

**Affiliations:** 1 Research Center for Implementation Nursing Science Initiative, Fujita Health University, Toyoake, Japan; 2 Department of Nursing, Akabane Central General Hospital, Kita-ku, Tokyo, Japan

**Keywords:** behavioral change, home care, knowledge, mobile application, pressure injury, mHealth, mobile health, apps, practice, injury, prevention, prototype, effectiveness, care manager, Japan, Pips-Map, questionnaire, wound care, pilot study, women

## Abstract

**Background:**

The use of mobile apps to promote knowledge level, practice, and behavioral change potential has become increasingly common. However, studies on apps targeting social welfare employees working in the home-care setting to prevent pressure injury (PI) are lacking. The care manager (CM) plays a key role in connecting the demand and supply of home-care services. PI is more prevalent in the home-care setting, where resources are limited, than in acute settings.

**Objective:**

The research hypothesis was that CMs who use a mobile app will have improved general knowledge and heightened practice for PI prevention, compared to that before using the app. This study aimed to assess the effectiveness of a PI prevention support mobile app prototyping model (Pips-Map) in improving the knowledge level, practice, and behavioral change potential of CMs in PI prevention in the home-care setting.

**Methods:**

This was conducted between December 2021 and December 2023 as a single-arm, pre-post pilot study including 27 CMs who worked in a Japanese city. Pips-Map was used for 6 months in daily practice, and a self-administered test questionnaire was used to assess participants’ knowledge and practice in PI prevention before or after using Pips-Map. At the end of the posttest, a validated App Behavior Change Scale was used to analyze behavioral change potential. This study followed the Consolidated Standards of Reporting Trials (CONSORT) extension to pilot and feasibility trials.

**Results:**

In total, 19 participants were analyzed. Out of 55 points, the total mean knowledge score significantly increased from 30.9 (SD 5.9) in the pretest group to 36.1 (SD 5.9) in the posttest group (*P*=.0003). The number of participants with a total score of >70% (adequate knowledge level) increased from 2 (11%) to 7 (36.8%), but the difference was not statistically significant (*P*=.07). For the level of practice, out of 21 points, the total score increased from 15.2 (SD 3.1) in the pretest group to 16.2 (SD 3.0) in the posttest group, but no statistically significant differences were observed (*P*=.16). The behavior change scale revealed that participants positively evaluated the Pips-Map to provide information on PI prevention guidelines but had concerns regarding inadequate usability and financial incentives of Pips-Map.

**Conclusions:**

The use of Pips-Map for 6 months in actual practice increased the knowledge level of Japanese CMs in PI prevention, but it did not change the level of practice. Considering the need for updating apps that aim to promote behavioral change, this study identified some limitations of Pips-Map. Thus, revisions must be made to adapt Pips-Map to home-based care needs.

## Introduction

### Backgrounds

Many developed countries are facing the challenge of a declining birthrate and aging population. Considering that Japan has the world’s most advanced aging population, the Japanese government has shifted the focus of health care from hospital- to home- or community-based care provided in the home-care setting [1]. To provide comprehensive community-based care, the Japan-specific geriatric care system (Community-based Integrated Care System, a medical- and social-welfare networking model) works with the Japan-specific long-term care insurance system. The care manager (CM), who possesses specialized knowledge necessary to help individuals in need of assistance or care to achieve more independent lives, plays a key role in connecting the demand and supply of long-term care services [2]. A workflow of CMs in home-based care services was summarized in a recent study [3]. The CM is responsible for providing care management services, including checking the physical condition and living circumstances of individuals, establishing and adjusting care plans according to individual needs, and monitoring individual care. To obtain a CM license, an individual must have ≥5 years of geriatric care experience as a medical professional (physician or nurse) and social welfare professional (care worker or social worker) and should have passed the examination. Compared to CMs with medical backgrounds, the number of CMs with social welfare backgrounds is higher, because obtaining a CM license is a career advancement step. Compared with CMs who studied medicine, those from the social welfare background may not have sufficient knowledge or practice regarding the prevention and treatment of chronic diseases, such as pressure injury (PI) [2].

Although PIs are pervasive in all age groups, most of them occur in older individuals with immobility and chronic diseases, such as heart disease, respiratory illness, and dementia [4]. PI can result in poor quality of life for patients and socioeconomic burden [5,6]. PI is more prevalent in the home-care setting, where resources are limited, than in acute settings, with prevalence rates of 1.76%‐26% [7-9]. Although there is broad consensus that some PIs are unavoidable, most are considered preventable [10]. A paper-based risk assessment tool for preventing PIs that can be used by Japanese CMs has recently been developed and implemented [11-13]; however, its application to digital technology is still under consideration.

### Prior Work

Digital technologies have become an emerging branch of medicine, with technology-based applications being used to improve disease knowledge and user activations being modified by relying on health-related information [14-16]. Mobile apps for PIs can be grouped into apps for PI treatment and apps for PI prevention. European wound management experts developed a clinical support mobile app based on a global wound care framework to implement care plans and promote wound healing [17]. Do Khac et al [18] evaluated a free-access mobile health app compared with standard technique to assess its reliability and validity of measurement of surface area of PIs in patients with spinal cord injury. In Japan, a PI treatment support mobile app was developed for sharing PI information with a multidisciplinary care team or for conducting remote consultation with wound care specialists [19,20]. A smartphone app focusing on PI prevention education was developed to evaluate the PI knowledge of informal carers and app acceptability and usability [21]. A guideline app was designed to present evidence-based guideline recommendations with easy access [22]. Amann et al [23] qualitatively investigated barriers and facilitators to the adoption of a self-management app for PI prevention in individuals with spinal cord injury. However, studies assessing a mobile app in improving knowledge and behavioral change within social welfare workers, such as CMs, who work at home-based care facilities, are lacking.

### Aim of This Study

The research hypothesis is that CMs who experience a mobile app will improve general knowledge and heightened practice about PI prevention compared to before the experience. The primary objective was to investigate the effects of CMs on improving knowledge level when using a PI prevention support mobile app prototyping model (Pips-Map), which has been developed by the industry. In addition to improving the knowledge level, we surveyed whether the practice level of CMs for PI prevention was heightened when using Pips-Map as a secondary objective. We further investigated to determine whether the use of the app promoted behavior change in PI prevention among CMs and which specific features of the app positively or negatively influenced the behavior change.

## Methods

### Prototyping Model

Pips-Map was designed and developed by ALCARE Co. Ltd. (Tokyo, Japan) to support research and practice in wound care, home-based care, etc. According to the developer, the key desktop design concept of Pips-Map was to facilitate information sharing among home health care providers with different specialties and change their behavior for preventing PI development in patients who received assistance or nursing care at home. Pips-Map is unique because it facilitates information sharing among home health care providers, including CMs, home-care nurses, and primary physicians for each home-based patient. Displaying products formulary for preventing PI in Pips-Map helps in selecting products for each patient in a timely manner. Pips-Map has 5 functions: (a) photographing skin using a standard smartphone or tablet camera; (b) assessing PI risk according to the Japanese Ohura-Hotta scale; (c) entering data on prophylactic planning or comments based on PI risk assessment; (d) displaying products (eg, pressure redistribution mattress, skin care products, prophylactic dressings) formulary; and (e) data sharing between medical and social welfare professionals for each patient. Pips-Map includes a software system named Medical Care Station, which was developed by Embrace Co., Ltd. (Tokyo, Japan), to provide patient information. The system is a security-conscious, completely private social networking service that shares information in a timeline format. Patient data are stored on the system but not linked to the electronic medical records of each site. Selected screenshots from Pips-Map are presented in Multimedia Appendix 1.

### Study Design

This prospective, single-arm, interventional pilot study was conducted at a university from December 2021 to December 2023. There were no major changes in study methods after the trial commencement. This study followed the Consolidated Standards of Reporting Trials (CONSORT) extension to pilot and feasibility trials [24] from the Equator Network, which assess the feasibility of conducting a future definitive randomized controlled trial rather than the effectiveness or efficacy of an intervention. As an editorial, Lancaster et al have provided some general guidance on how to report non-randomized pilot and feasibility studies, including a pre-post test, using the CONSORT extension to pilot and feasibility trials [25]. The checklists for the reporting guideline in this study were provided in Multimedia Appendix 2.

### Setting and Sample

The accessible population included individuals with a CM license and those who participated in the “Kita-ku Care Managers’ Association” in Kita-ku, Tokyo (n=185). According to statistics from the Japanese Ministry of Health, Labor and Welfare in the year 2022, the number of CMs working full-time at home-care support facilities is 99,220. Of these, 8,943 live in Tokyo [26]. Kita-ku lies in the northeastern region of Tokyo and is one of the 23 districts. The Kita-ku Care Managers’ Association works in Kita-ku, and the number of CMs accounts for approximately 2.1% (185/8943) of them in Tokyo. Japanese CMs could use an Android- and iOS-based mobile application. As power analysis could not be performed, this study was considered a pilot study. Pilot studies are needed to obtain preliminary data for calculating sample size for the primary outcome [27].

### Study Procedure

Kita-ku Care Managers’ Association holds regular meetings for information exchange and acquisition of knowledge about home-care management. In this meeting, we orally explained the details of this study after obtaining agreement for cooperation with this study from the member of the executive committee of the Kita-ku Care Managers’ Association. A total of 27 individuals agreed to participate, and the pretest was completed. Pips-Map was downloaded and operated for 6 months in daily practice. The participants were instructed on how to use Pips-Map and record the details of PI prevention in Pips-Map through web-based guidance. Technical support on how to use Pips-Map was provided by the researcher through telephone and e-mail.

A pretest or posttest was performed to measure participants’ knowledge level and practice level in PI prevention. The pretest was conducted on the same day when informed consent was obtained, and the posttest was conducted within a month after using Pips-Map. At the end of the posttest, an additional questionnaire survey was conducted to assess Pips-Map to promote behavioral change potential. All tests and questionnaires were made available via web-based Google Forms. The completion of pretest and posttest and additional questionnaire required approximately 20 and 10 minutes, respectively.

### Measurement

#### General Characteristics of Participants

The language used for the questionnaire was Japanese. The questionnaire was divided into 4 parts. Part 1 contained general characteristics of participants, including age range, sex, years of experience as a CM, professional background, and education level.

### Primary Outcome

In part 2, participant knowledge on PI prevention was evaluated. Although 2 validated knowledge tests for PI prevention have been reported [28,29], most respondents in these studies were medical professionals, such as nurses who worked at hospitals. The test questions were difficult to answer for social welfare professionals such as Japanese CMs. Therefore, the current self-administered test questionnaire was developed via collaboration between a nurse with certification in wound, ostomy, and continence working at the home-care division of a hospital and a senior assistant professor certified in wound, ostomy, and continence nursing. For questionnaire development, they referred to a previous report [30] and the Japanese PI guideline created by the Japanese Society of Pressure Ulcers [31]. This part consisted of 55 test questions reflecting 6 themes: (1) anatomy, (2) risk assessment, (3) pressure, (4) friction and shear, (5) moisture, and (6) nutritional support. Answering options and scoring rules varied depending on items. For items 1‐4, 7, and 11‐13, the participants were instructed to select “true,” “false,” or “I do not know.” Each correct answer was assigned one point, and no points were assigned to wrong answers and “I do not know” answers. For items 5, 9, and 14, the participants selected “know” or “do not know.” A score of 1 was assigned for “know” and 0 for “do not know.” For items 6, 8, and 10, the multiple-choice questions had several options along with the “I do not know” option. A correct answer was assigned a score of 1, and an incorrect answer was assigned 0. The maximum score was 55 (Multimedia Appendix 3).

### Secondary Outcomes

In part 3, the participants were asked about the practice of preventive strategies for PIs. This part consisted of 21 test questions reflecting 6 sections: (1) risk assessment, (2) pressure, (3) friction and shear, (4) moisture, (5) nutritional support, and (6) communication with other professionals. The test questionnaire in this section asked whether the participants implemented those actions or not. The answer choice for each question was “practice” or “do not practice.” One or zero point was given when the participant answered “practice” or “do not practice,” respectively. The maximum practice score was 21 in this section (Multimedia Appendix 4).

In part 4, behavioral change potential was measured using a validated App Behavior Change Scale (ABACUS) [32] with a minor modification. For the modification, the item “does the app provide a material or social reward or incentive?” in the original paper was divided into the following 2 items: “does the app provide a financial incentive?” and “does the app provide a material or social reward?” The scale comprised 22 questions regarding a determinant of behavioral change potential of mobile apps, each on a 5-point Likert scale (1=“strongly disagree” to 5=“strong agree”) with total score 22‐110. A higher score indicated higher levels of promoting behavioral change by app usage. Previous literature showed that potential behavioral change of users of apps was evaluated by ABACUS in a wide range of research fields, including depression, diabetes, osteoarthritis, and physiotherapy care [33,34].

### Data Analysis

For general characteristics, all data were categorical variables and expressed as frequencies and percentages. For level of knowledge, as a primary outcome, a dependent *t*-test (for continuous variables), the Wilcoxon signed-rank test (for ordinal variables), and McNemar’s test (for categorical variables) were conducted to compare differences between each item and total score between the pretest and posttest groups. Ordinal variables with ≥5 categories were treated as continuous variables. A study on the learning outcome criteria for PI prevention considered an average total score of >70% satisfactory [35]. As a secondary outcome, similar analysis was conducted for the level of practice. For behavioral change potential, mean and SD were reported in each item and total score of ABACUS. All statistical analyses were conducted using the Statistical Package for the Social Sciences version 28.0 (IBM Corporation, Armonk, NY, USA). *P* values of <.05 were considered to indicate statistical significance.

### Ethical Considerations

This study was approved by the university’s ethics review board (approval number HM21-335). Electronic informed consent was obtained from all participants before participation. All experiments in this research were performed according to the principles of the Declaration of Helsinki. Participant data were kept confidential. This study has been registered in the Clinical Trials Registry (UMIN Clinical Trial Registry number: UMIN000048904) and is accessible online. Each participant who completed the questionnaire was given a gift certificate (5000 JPN yen) as a financial reward.

## Results

### General Characteristics of Participants

**Figure 1.**
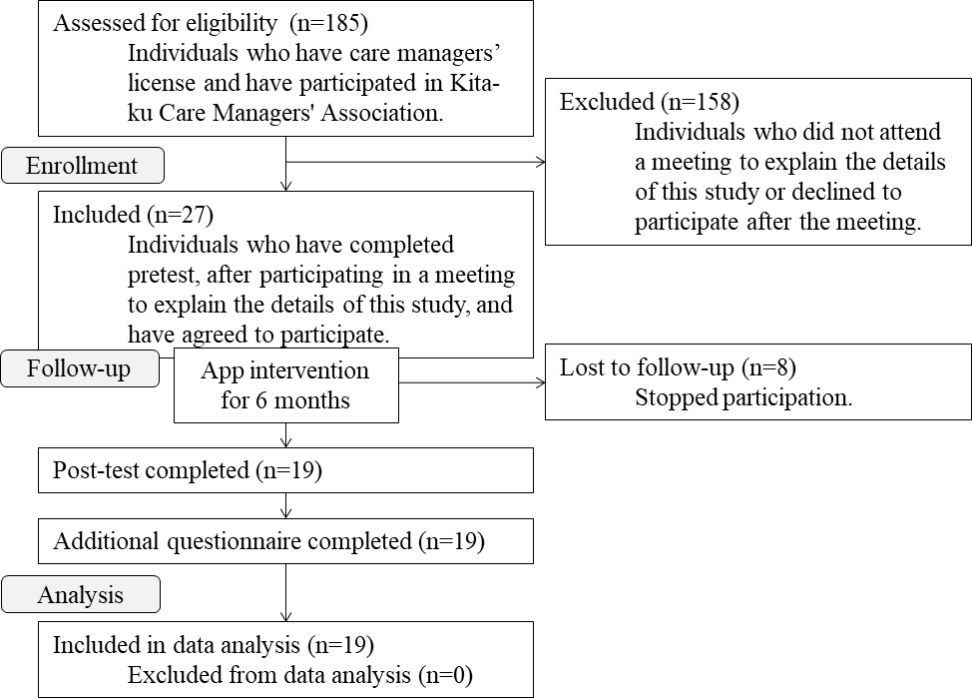
Flowchart of participant progression in pretest and posttest study. A total of 27 care managers who participated in the “Kita-ku Care Managers’ Association” in a district in Tokyo agreed to participate. As an intervention, a pressure injury prevention support mobile app prototyping model was operated for 6 months in daily practice. A pretest or posttest was performed to measure participants’ knowledge level and practice level in pressure injury prevention. After the posttest was completed, an additional questionnaire survey was conducted to assess the mobile app to promote behavioral change potential. In total, 19 participants were included in data analysis. This study was conducted from December 2021 to December 2023.

In total, 19 participants were analyzed (Figure 1). The recruitment of participants for this study trial started on September 11, 2022, and the follow-up was completed on August 31, 2023. Most participants were in their 40 and 50 seconds, and 12 out of 19 participants (63%) were female. All of the participants had a background in social welfare; that is, participants who studied medicine or nursing were not included in this study (Table 1).

**Table 1.** General characteristics of participants. Under the Japan-specific long-term care insurance system, to obtain a care manager license, an individual must have ≥5 years of geriatric care experience as a medical professional (physician or nurse) and social welfare professional (care worker or social worker). Compared with care managers who studied medicine, those from the social welfare background may not have sufficient knowledge or practice regarding the prevention of pressure injury. All of the care managers who participated in this study had a professional background in social welfare, not medicine. As the primary outcome included knowledge levels of pressure injury prevention, the educational level for the study participants was an important characteristic to be confirmed.

Characteristics	Overall (N=19)
Age, n (%)	
30s	3 (16)
40s	7 (37)
50s	6 (32)
60s	3 (16)
Sex, n (%)	
Male	7 (37)
Female	12 (63)
Years of experience as a care manager, n (%)	
<5	3 (16)
≥5 and <10	4 (21)
≥10	12 (63)
Professional background, n (%)	
Social welfare	19 (100)
Medical	0 (0)
Education level, n (%)	
Junior high school	1 (6)
High school	8 (44)
Vocational school	5 (28)
Junior college	1 (6)
College	3 (17)

### Primary Outcome Evaluation

As shown in Table 2, the total score of knowledge significantly increased in the posttest group compared with that in the pretest group (*P*=.0003). The number of participants with a total score >70% increased from 2 (11%) in the pretest group to 7 (37%) in the posttest group, but the differences were not statistically significant (*P*=.07). In each test questionnaire, “anatomy,” “risk assessment,” and “pressure” increased significantly, but the remaining items were similar between the 2 groups.

**Table 2.** Comparison of knowledge level between the pretest and posttest groups. for the questionnaire development to evaluate the participants’ knowledge level in pressure injury prevention, the authors referred to a previous report [28] and the Japanese pressure injury guideline [29]. It consisted of 55 test questions reflecting 6 themes: (1) anatomy, (2) risk assessment, (3) pressure, (4) friction and shear, (5) moisture, and (6) nutritional support. The maximum score was 55. A pretest or posttest was performed to measure participants’ knowledge level in pressure injury prevention. The pretest was conducted on the same day when informed consent was obtained, and the posttest was conducted within a month after intervention. The total score of knowledge significantly increased in the posttest group compared with that in the pretest group. Statistical analysis was conducted via those listed in footnotes b, d, and e.

Questionnaire	Pretest	Posttest	*P*-value
Anatomy, mean (SD^a^)	16.2 (2.6)	19.2 (3.6)	.001^b^
Risk assessment, mean (SD)	0.3 (0.5)	1.2 (1.4)	.009^b^
Pressure, mean (SD)	6.3 (2.0)	7.4 (1.5)	.013^b^
Friction and shear, mean (SD)	4.7 (1.6)	4.8 (1.3)	.76^b^
Moisture, median (IQR^c^)	2 (2–3)	3 (2–3)	.25^d^
Nutritional support, mean (SD)	1.2 (0.9)	1.1 (1.3)	.56^b^
Total score, mean (SD)	30.9 (5.9)	36.1 (5.9)	.0003^b^
Total score>70%, n (%)	2 (11)	7 (37)	.070^e^

a SD, standard deviation.

b dependent *t*-tests for continuous variables.

c IQR, interquartile range.

d Wilcoxon signed-rank test for ordinal variables, and.

e McNemar’s test for categorical variables.

### Secondary Outcome Evaluation

As shown in Table 3, the total score of practice increased from 15.2 (SD 3.1) in the pretest group to 16.2 (SD 3.0) in the posttest group, but no statistically significant differences were observed (*P*=.16). The score of “risk assessment” increased significantly (*P*=.030). The average total score for behavioral change potential using ABACUS was 71.04 (SD 11.55) (Multimedia Appendix 5). The highest score was achieved for the question “was the app created with expertise and does the app provide information consistent with national guidelines?” Lower scores (mean value of <3.0) were obtained for the questions “does the app provide financial incentives for the users and their organization?,” “does the app have the ability to export data from app?,” and “does the app allow or encourage practice or rehearsal, in addition to daily activities?”

**Table 3.** Comparison of level of practice between the pretest and posttest groups. The participants were asked about the practice of preventive strategies for pressure injuries. It consisted of 21 test questions reflecting 6 sections: (1) risk assessment, (2) pressure, (3) friction and shear, (4) moisture, (5) nutritional support, and (6) communication with other professionals. The maximum practice score was 21. The pretest was conducted on the same day when informed consent was obtained, and the posttest was conducted within a month after the intervention. No statistically significant differences were shown in the total score of practice between the posttest and posttest groups. Statistical analysis was conducted via a, c, and e.

Questionnaire	Pretest	Posttest	*P* value
Risk assessment, n (%)	4 (21)	6 (32)	.48^a^
Pressure, mean (SD^b^)	6.8 (2.0)	7.9 (1.7)	.030^c^
Friction and shear, median (IQR^d^)	2 (1–2)	2 (1–2)	.74^e^
Moisture, median (IQR)	2 (2–2)	2 (1–2)	.60^e^
Nutritional support, mean (SD)	2.7 (1.0)	2.6 (1.0)	.84^c^
Communication with other professionals, median (IQR)	2 (2–3)	2 (2–2)	.60^e^
Total score, mean (SD)	15.2 (3.1)	16.2 (3.0)	.16^c^
Total score>70%, n (%)	10 (53)	11 (58)	.68^a^

a McNemar’s test for categorical variables.

b SD, standard deviation.

c dependent *t*-tests for continuous variables, and.

d IQR, interquartile range.

e Wilcoxon signed-rank test for ordinal variables.

## Discussion

### Principal Findings

The use of a mobile app provides a more engaging and accessible approach to clinical practice at home care, fitting well with the evolving demands of the digital ages. The development of a PI prevention support mobile app and its evaluation as an educational tool has already been reported; however, studies approaching a mobile app in improving knowledge, practice, and behavioral change within social welfare workers who work at home-care facilities are lacking. In this study, we used Pips-Map in an actual clinical setting, and the effect of improving general knowledge, heightened practice, and positively changing behavior among CMs in the prevention of PI development was investigated in a pretest-posttest study design. As the incidence of PI in the home-care setting impacts the quality of care provided, the adoption of good practice with adequate knowledge through improvement in PI prevention is necessary. Adequate knowledge is essential for assessing behavioral change. The key finding of this study is that the use of a mobile app in daily practice can increase knowledge about PI prevention among Japanese CMs. However, our results could not demonstrate that increased knowledge indicated improved practice level.

The most significant finding of this study is that Pips-Map is effective in improving the total score of knowledge level (Table 2). Consistently, a 6-week interventional study based on a mobile app focused on PI prevention education for informal carers of individuals at risk of PI [21]. Using a self-administered PI knowledge assessment questionnaire consisting of 4 categories (support surfaces, nutrition and hydration, keep moving, and skin care and inspection), they reported that the use of the app effectively increased knowledge level regarding PI prevention. CMs need to manage their care work with other responsibilities, and their busy schedule makes learning about PI prevention challenging. Active methodology with digital technologies enables flexibility for conducting these activities once the barrier of time and space has been overcome. Compared with conventional on-site education, such as through lectures or handouts, Pips-Map is more advantageous as a training tool in terms of accessibility, leading to the prevention of common PIs in the home-care setting.

However, changes in the participants’ PI prevention practices were not observed (Table 3). This might be explained by our results to understand the behavioral change potential using ABACUS (Multimedia Appendix 5). A high score was obtained for item 2, “providing information consistent with national guidelines,” which directly affects the increase in the knowledge of PI prevention. By contrast, lower scores were observed for item 13, “ability to export data from app,” item 14, “providing a financial incentive,” and item 19, “allowing for practice or rehearsal,” which are significantly important items for CMs. In this current form, we believe that Pips-Map is didactic and inadequate in terms of customized content and usability.

As shown in the lowest mean score in ABACUS, the participants did not perceive receiving social or financial incentive by using the app. Although financial rewards are a powerful extrinsic motivation for app use, studies suggest that the most powerful plans for behavioral change include both extrinsic and intrinsic factors. For example, rankings, social recognition, and gaming are reported to be strong intrinsic factors relating to long-term app use [36]. In addition, the opinions raised from the behavioral change questionnaire regarding customized content and usability could not allow for adaptation of the app in the real-world setting. Hence, we will provide feedback to the industry on the need to create a modified version of the app to address these issues for the development phase. In addition, we will investigate the impact of the modified version on behavioral change of the user and health care outcomes of individuals who need assistance or nursing care at home. Evidence on knowledge and practice of wound care is updated daily, and all the researchers have to keep up with the fast-moving pace of app technology.

### Limitations

Our study has several limitations. First, our tests for knowledge and practice of PI prevention have not yet been validated. It would be better to use validated tests simultaneously; however, tests targeting Japanese CMs are lacking. The face validity, construct validity, and reliability of the knowledge and practice test questions used in this study were not considered, because we prioritized evaluating the effect of a mobile app prototype on knowledge, practice, and behavior change. As a future work, determining the reliability and validity of the newly developed test questions is a key imperative, which ultimately could benefit individuals who receive preventive care as well as social welfare professionals such as CMs who provide it.

Next, this single-arm, pre-post study aimed to preliminarily assess the effectiveness and feasibility of Pips-Map. Determining whether the value of the outcome observed upon intervention is better than the value without intervention is challenging. Finally, we did not perform power analysis to estimate the ideal sample size because an accurately estimated population could not be set before the start of this study. The sample size is relatively small, but it should be acceptable considering the design of a pilot study. The authors were unable to determine the cause for the dropout of study participants because it was difficult to chase them. For the effectiveness and usability of modified Pips-Map, comparative studies between intervention and nonintervention groups with appropriate sample sizes are required in the future.

### Conclusions

Our results revealed that the use of a mobile app prototyping model for 6 months in actual practice increased the knowledge level of Japanese CMs in preventing PI development, but it did not affect the level of practice between the pretest and posttest groups. Considering the need for updating apps that aim to promote behavioral change, this study identified limitations associated with the app. A higher quality app must be developed that is suitable for the home-care setting.

## Supplementary material

10.2196/57768 Multimedia Appendix 1 Concept of the pressure injury prevention support mobile application prototyping model (Pips-Map).

10.2196/57768 Multimedia Appendix 2 CONSORT 2010 checklist of information to include when reporting a pilot or feasibility trial.

10.2196/57768 Multimedia Appendix 3 Pressure injury prevention knowledge test questionnaire.

10.2196/57768 Multimedia Appendix 4 Pressure injury prevention practice test questionnaire.

10.2196/57768 Multimedia Appendix 5 Results of app behavioral change scales.

## Abbreviations

ABACUS: App Behavior Change Scale
CM: care manager
CONSORT: Consolidated Standards of Reporting Trials
PI: pressure injury
Pips-Map: pressure injury prevention support mobile app prototyping model
