# Supplementary figures and images for: Evaluating the Knowledge Level, Practice, and Behavioral Change Potential of Care Managers in Pressure Injury Prevention Using a Mobile App Prototyping Model in the Home-Care Setting: Single-Arm, Pre-Post Pilot Study
Source: JMIR Form Res. 2025 Feb 7;9:e57768. doi: 10.2196/57768 (PMC11830480; doi:10.2196/57768)

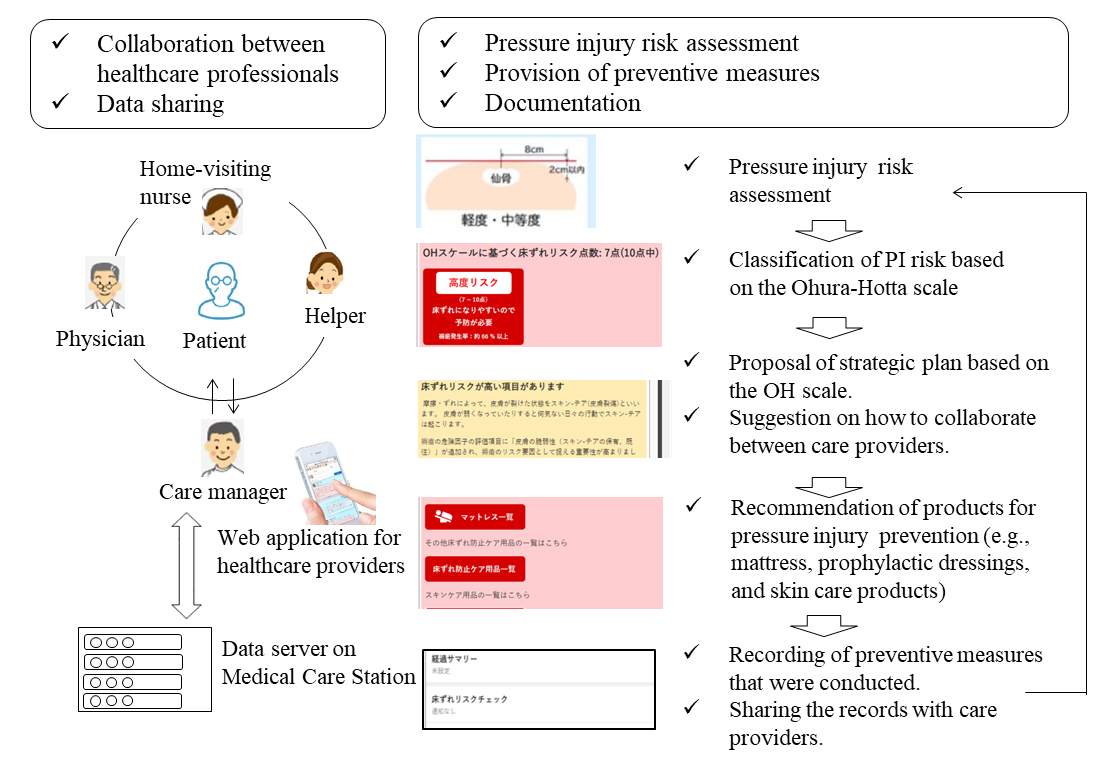

Supplement: Multimedia Appendix 1 [file formative-v9-e57768-s001.png]
