# Supplementary material for: Evaluating the Knowledge Level, Practice, and Behavioral Change Potential of Care Managers in Pressure Injury Prevention Using a Mobile App Prototyping Model in the Home-Care Setting: Single-Arm, Pre-Post Pilot Study
Source: JMIR Form Res. 2025 Feb 7;9:e57768. doi: 10.2196/57768 (PMC11830480; doi:10.2196/57768)
Supplement: Multimedia Appendix 3 [file formative-v9-e57768-s003.doc]

Appendix 2. Pressure injury prevention knowledge test questionnaire.

| Questionnaire (answer) | | Scoring rules | Allotment of points |
| --- | --- | --- | --- |
| Anatomy | |  |  |
|  | 1. In the lateral position, PIs are likely to develop on the ... a. ear (true) b. shoulder (true) c. greater trochanter (true)  d. thigh (false) e. knee (true) f. malleolus (true) | For each skin site, correct = 1, incorrect or do not know = 0 | 6 |
|  | 2. In the Fowler’s position, PIs are likely to develop on the ... a. shoulder blade (true) b. sacrum (true) c. ischial tuberosity (true) d. thigh (false) e. calf (false) f. heel (true) | For each skin site, correct = 1, incorrect or do not know = 0 | 6 |
|  | 3. In the sitting position, PIs are likely to develop on the ... a. shoulder blade (true) b. elbow (true) c. sacrum (true) d. ischial tuberosity (true) e. thigh (false) f. heel (true) | For each skin site, correct = 1, incorrect or do not know = 0 | 6 |
|  | 4. In the supine position, PIs are likely to develop on the ... a. heel (true) b. thigh (false) c. sacrum (true) d. elbow (true) e. shoulder blade (true) f. back of head (true) | For each skin site, correct = 1, incorrect or do not know = 0 | 6 |
| PI risk assessment | |  |  |
|  | 5. For PI risk assessment scales, I know the ... a. Braden scale b. OH scale c. K scale d. Japanese government scale | For each scale, yes = 1, no = 0 | 4 |
| Pressure | |  |  |
|  | 6. In individuals at high risk of developing PIs, the position should be changed frequently and at least every ... a. 1 h b. 2 h (correct) c. 3 h d. 4 h e. 5 h f. do not know | Correct = 1, incorrect = 0 | 1 |
|  | 7. A preventive measure to reduce the magnitude of pressure in the lateral position is ... a. Side-lying in a lateral tilt position at 30° (true) b. Using a pressure-reducing cushion under bone prominence sites (true) c. Distributing the individual's weight over a smaller surface area (false) | For each question, correct = 1, incorrect or do not know = 0 | 3 |
|  | 8. For individuals in a sitting position or on a wheelchair, it is better to reseat every … a. 15 min (correct) b. 30 min c. 1 h d. 2 h e. 3 h f. do not know | Correct = 1, incorrect = 0 | 1 |
|  | 9. Regarding resitting to maintain low pressure distribution, I know the following measure is correct. a. Patients themselves lean forward and raise their buttocks up b. Use arm support, put their feet on the floor, and raise their buttocks up (in case of using a wheelchair) c. Lean to one side and then lean to the other side d. Caregiver lifts up the patients' body and then lifts their buttocks e. Change tilt and recline angles | For each question, yes = 1, no = 0 | 5 |
| Friction and shear | |  |  |
|  | 10. When elevating the head-of-bed, the appropriate head-of-bed angle to minimize shear forces is ... a. 30° (correct) b. 45° c. 60° d. 90° e. do not know | Correct = 1, incorrect = 0 | 1 |
|  | 11. When elevating the head-of-bed, the type of bed that can minimize shear forces is ... a. With two electric motors (false) b. With three electric motors (true) | For each question, correct = 1, incorrect or do not know = 0 | 2 |
|  | 12. When elevating the head-of-bed, the appropriate preventive measure is... a. To shift individual's body slightly toward the head-of-bed (false) b. To match the body's bending points with those of the bed (true) c. To elevate the bed at the feet and then the head (true) d. To elevate the bed at the head and then the feet (false) e. To insert the care provider's hand equipped with sliding gloves between the individuals' body and mattress (true) | For each question, correct = 1, incorrect or do not know = 0 | 5 |
| Excess moisture due to incontinence | |  |  |
|  | 13. When changing diapers in individuals with incontinence, the appropriate PI preventive measure is ... a. To wash the genital and surrounding sites with soap each time (false) b. To use a superabsorbent pad (true) c. To apply skin protector or ointment around the genital skin (true) | For each question, correct = 1, incorrect or do not know = 0 | 3 |
| Nutritional support | |  |  |
|  | 14. As clinical indicators associated with nutritional status for PI prevention, I know... a. Unintended weight loss b. Insufficient dietary intake c. Mini nutritional assessment (MMA) d. Controlling nutritional status (CONUT) e. Serum albumin level  f. Subjective Global Assessment (SGA) | For each indicator, yes = 1, no = 0 | 6 |

Abbreviations: PI, pressure injury
