# Supplementary material for: Evaluating the Knowledge Level, Practice, and Behavioral Change Potential of Care Managers in Pressure Injury Prevention Using a Mobile App Prototyping Model in the Home-Care Setting: Single-Arm, Pre-Post Pilot Study
Source: JMIR Form Res. 2025 Feb 7;9:e57768. doi: 10.2196/57768 (PMC11830480; doi:10.2196/57768)
Supplement: Multimedia Appendix 4 [file formative-v9-e57768-s004.doc]

Appendix 3. Pressure injury prevention practice test questionnaire.

| Questionnaire (answer) | | Scoring rules | Allotment of points |
| --- | --- | --- | --- |
| PI risk assessment | |  |  |
|  | 1. In daily work, you use a PI risk assessment scale named... a. Braden scale b. OH scale c. K scale d. MHLW scale | Selected one of the scales = 1, no = 0 | 1 |
| Pressure | |  |  |
|  | 2. When choosing the type of wheelchair, I consider … a. Range of motion of hip joints in wheelchair users b. Rounding of the back of wheelchair users c. Pressure distribution based on the wheelchair users' body shape and posture d. Sheet width e. Sheet depth and height f. Foot and arm supports | For each question, yes = 1, no = 0 | 7 |
|  | 3. In daily work, I usually … a. recommend the use of a wheelchair cushion to prevent PI development b. request to select professionals who have knowledge of seating, in case of selecting wheelchair cushions for individuals who are unable to sit in a kneeling position or have paralysis or sensory impairment c. request the involvement of other professionals to provide appropriate seating for individuals who are unable to sit up on their own or have poor sitting posture | For each question, yes = 1, no = 0 | 3 |
| Friction and shear | |  |  |
|  | 4. During head-of-bed elevation for individuals who are bedridden or have poor mobility in daily work, I usually … a. recommend that healthcare workers use sliding sheets or assistance gloves b. request the involvement of rehabilitation by physical therapists or other professionals | For each question, yes = 1, no = 0 | 2 |
| Excess moisture due to incontinence | |  |  |
|  | 5. Regarding diaper use in individuals with urinary and/or fecal incontinence, I usually … a. recommend the individual or care provider to use highly absorbent pads b. propose that the individual or care provider should establish ways of putting a diaper on | For each question, yes = 1, no = 0 | 2 |
| Nutritional support | |  |  |
|  | 6. In daily work, I usually … a. recommend individuals or care providers to ensure having or providing foods that contain protein at every meal as a side dish b. recommend individuals or care providers to ensure having or providing foods that are rich in protein at every meal as a side dish, if the nutritional intake is poor c. consider requesting a home-care based doctor or nurse to prescribe enteral nutrition when the food and fluid intake is inadequate | For each question, yes = 1, no = 0 | 3 |
| Communication with other professionals | |  |  |
|  | 7. To provide correct advice to individuals or their family in PI prevention, I usually work in cooperation with... a. a nurse who is an expert in skin assessment, comprehensive preventive measures, and early detection of PI development b. a rehabilitation staff who is an expert in mobility involvement and position change c. a nutrition staff who is an expert in nutritional assessment and preventive involvement. | For each question, yes = 1, no = 0 | 3 |

Abbreviations: PI, pressure injury
