# Supplementary material for: Evaluating the Knowledge Level, Practice, and Behavioral Change Potential of Care Managers in Pressure Injury Prevention Using a Mobile App Prototyping Model in the Home-Care Setting: Single-Arm, Pre-Post Pilot Study
Source: JMIR Form Res. 2025 Feb 7;9:e57768. doi: 10.2196/57768 (PMC11830480; doi:10.2196/57768)
Supplement: Multimedia Appendix 5 [file formative-v9-e57768-s005.doc]

Appendix 4. Results of app behavioral change scales.

|  | | Mean | SD |
| --- | --- | --- | --- |
|  | |  |  |
| Knowledge and information | |  |  |
| 1 | Does the app have the ability to customize and personalize some features? | 3.11 | 0.74 |
| 2 | Was the app created with expertise and/or does the app provide information consistent with national guidelines? | 4.26 | 0.65 |
| 3 | Does the app ask for baseline information? | 3.68 | 0.82 |
| 4 | Does the app provide instruction on how to perform the behavior? | 3.53 | 1.02 |
| 5 | Does the app provide information about the consequences of continuing and/or discontinuing behavior? | 3.68 | 0.82 |
| Goals and planning | |  |  |
| 6 | Does the app ask for willingness for behavior change? | 3.37 | 0.96 |
| 7 | Does the app allow for the setting of goals? | 3.47 | 1.02 |
| 8 | Does the app have the ability to review goals, update, and change when necessary? | 3.47 | 1.02 |
| Feedback and monitoring | |  |  |
| 9 | Does the app provide the user the ability to rapidly and easily understand the difference between current action and future goals? | 3.58 | 0.96 |
| 10 | Does the app have the ability to allow the user to easily self-monitor behavior? | 3.63 | 0.96 |
| 11 | Does the app have the ability to share behaviors with others (including social media or forums) and/or allow for social comparison? | 3.11 | 1.10 |
| 12 | Does the app have the ability to provide user feedback—either from a person or automatically? | 3.79 | 0.92 |
| 13 | Does the app have the ability to export data from the app? | 2.42 | 1.12 |
| 14 | Does the app provide a financial incentive? | 1.63 | 1.01 |
| 15 | Does the app provide a material or social reward? | 3.16 | 1.01 |
| 16 | Does the app provide general encouragement? | 3.47 | 0.77 |
| Actions | |  |  |
| 17 | Does the app have reminders and/or prompts or cues for activity? | 3.53 | 1.02 |
| 18 | Does the app encourage positive habit formation? | 3.74 | 0.99 |
| 19 | Does the app allow or encourage practice or rehearsal, in addition to daily activities? | 2.95 | 1.31 |
| 20 | Does the app provide opportunity to plan for barriers? | 3.63 | 0.96 |
| 21 | Does the app assist with or suggest restructuring the physical or social environment? | 3.58 | 0.90 |
| 22 | Does the app assists with distraction or avoidance? | 3.74 | 0.99 |
| Overall | | 3.38 | 0.55 |
| Total score | | 74.53 | 12.15 |

Abbreviation; SD, standard deviation
